# Oxidative Stress-Induced Overactivation of Frog Eggs Triggers Calcium-Dependent Non-Apoptotic Cell Death

**DOI:** 10.3390/antiox11122433

**Published:** 2022-12-09

**Authors:** Alexander A. Tokmakov, Yudai Morichika, Ryuga Teranishi, Ken-Ichi Sato

**Affiliations:** 1 Institute of Advanced Technoogy, Faculty of Biology-Oriented Science and Technology, KinDai University, 930 Nishimitani, Kinokawa City 649-6493, Japan; 2 Laboratory of Cell Signaling and Development, Faculty of Life Sciences, Kyoto Sangyo University, Kyoto 603-8555, Japan

**Keywords:** oxidative stress, *Xenopus laevis*, eggs, overactivation, cell death

## Abstract

Excessive activation of frog eggs (overactivation) is a pathological process that renders eggs unfertilizable. Its physiological inducers are unknown. Previously, oxidative stress was shown to cause time- and dose-dependent overactivation of *Xenopus laevis* frog eggs. Here, we demonstrate that the oxidative stress-induced egg overactivation is a calcium-dependent phenomenon which can be attenuated in the presence of the selective calcium chelator BAPTA. Degradation of cyclin B2, which is known to be initiated by calcium transient in fertilized or parthenogenetically activated eggs, can also be observed in the overactivated eggs. Decline in mitochondrial membrane potential, ATP depletion and termination of protein synthesis manifest in the eggs within one hour of triggering overactivation. These intracellular events occur in the absence of caspase activation. Furthermore, plasma membrane integrity is compromised in the overactivated eggs, as evidenced by ATP leakage and egg swelling. In sum, our data demonstrate that oxidative stress-induced overactivation of frog eggs causes fast and dramatic disruption of cellular homeostasis, resulting in robust and expedited cell death by a calcium-dependent non-apoptotic mechanism.

## 1. Introduction

Oocytes and eggs of the African clawed frog *Xenopus laevis* are widely used in cell cycle and reproductive studies due to their high biochemical and cytological tractability. The fully grown oocyte and eggs are large cells, exceeding 1 mm in diameter. They can be obtained in abundance from living female frogs and easily maintained in vitro as a primary cell culture. Notably, oocytes and eggs are functionally different cells. The fully grown immature frog oocytes are not competent for fertilization. They are arrested at the diplotene stage of the first meiotic prophase, which is characterized by a low activity of the main meiotic regulators, the cytostatic factor (CSF) and maturation-promoting factor (MPF) [1,2]. MPF was originally defined by Masui and Markert as a cytoplasmic capacity from mature oocytes that causes complete maturation upon injection into immature oocytes, and CSF, as a cytoplasmic capacity from unfertilized eggs that promotes metaphase arrest upon transfer to early dividing embryos. It was later found that MPF represents a complex of cyclin B and Cdk1 kinase [3], and CSF was identified as a multicomponent system comprising the meiotic protein kinase Mos and the MAPK pathway [4,5,6]. Notably, the key meiotic determinant, Mos protein, is only transiently present during meiosis and it completely vanishes soon after fertilization [7,8]. Hormonal stimuli trigger meiotic oocyte maturation (i.e., meiosis) and ovulation, resulting in release of fertilization-competent mature oocytes from the ovaries and ovarian follicles. After the completion of maturation, ovulated oocytes are arrested at the metaphase of the second meiotic division due to the high activity of CSF and MPF. In maturing frog oocytes, the two factors are embedded in a loop of positive feedback [9] and their activities change in a coordinated and synchronous manner [10]. In frogs, the term “eggs” is conventionally used for mature metaphase II-arrested oocytes. The meiotic metaphase arrest in eggs prevents cell cycle progression and parthenogenesis before fertilization.

A dramatic difference exists between oocytes and eggs in regard to their durability. The immature fully grown *Xenopus* oocytes can reside in the ovaries and maintain their functional activity (i.e., responsiveness to ovulation hormones) for many months. On the other hand, ovulated frog and mammalian eggs can be successfully fertilized only within several hours to days following ovulation. In the absence of fertilization, ovulated mammalian eggs gradually deteriorate in the process of postovulatory aging, undergo fragmentation and eventually degrade by apoptosis [11,12,13]. Similarly, unfertilized *Xenopus* eggs spontaneously exit the metaphase II arrest and degrade by a well-ordered apoptotic process, both in external aquatic environments and in the genital tract, within 48 h after ovulation [14,15]. In contrast, the prophase arrested fully grown immature frog oocytes are markedly resistant to apoptosis [14]. It is widely recognized that spontaneous activation of eggs from different species renders them unfertilizable [16,17,18]. At present, physiological inducers of the spontaneous activation remain unidentified.

It is well established that the oxidative stress imposed on cells by reactive oxygen species (ROS) exerts multiple adverse effects and promotes various negative outcomes, such as accelerated cell ageing, apoptosis, tumorigenesis, etc. It was shown that high levels of intracellular ROS promote tumorigenesis via mutational damage and epigenetic modifications of mitochondrial and nuclear DNA [19,20]. The major producers of endogenous ROS in different cells, including cancer cells, are mitochondria and NADPH oxidases [20,21]. Both the contributors were found to be functionally active in *Xenopus* oocytes and eggs [21]. It was demonstrated that various reactive oxygen species, such as hydrogen peroxide, superoxide anion, hydroxyl radical and others, can impede quality of metaphase II-arrested mouse oocytes [22,23]. In mammalian oocytes, oxidative stress causes alterations in the structure of the metaphase spindle, interferes with the meiotic cell cycle and induces morphological features of apoptosis [13,24,25]. It was shown that increased levels of ROS can bring about aging-dependent spontaneous activation of *in vitro*-cultured postovulatory rat eggs [26]. Also, it was reported that hydrogen peroxide elevates intracellular calcium in *Xenopus* frog eggs, resulting in Src kinase-dependent egg activation [27]. It was further demonstrated that excessive treatment with hydrogen peroxide triggers egg overactivation, a phenomenon which gives rise to a very distinct egg phenotype [27,28]. Fast and irreversible cortical contraction, lipofuscin accumulation, depletion of intracellular ATP, decrease in the content of soluble cytoplasmic protein were found to occur in the frog eggs overactivated by strong oxidative stress [28]. Still, at present, the intracellular processes that take place in the overactivated eggs have not been investigated in detail. It was suggested that overactivated eggs die by a distinct, sequential and ordered, non-apoptotic mechanism.

In the present study, oxidative stress-induced overactivation of *Xenopus* eggs was further investigated. Here, we demonstrate that it is a calcium-dependent process, which can be attenuated in the presence of calcium chelators. Egg overactivation causes fast and dramatic disruption of cellular homeostasis, as witnessed by decline in mitochondrial membrane potential (MMP), depletion of intracellular ATP, termination of protein synthesis and breach of plasma membrane integrity. These events unfold in the absence of caspase activation, and they occur much faster than the hallmark events of the classical apoptotic process described previously in *Xenopus* eggs [14,15]. Thus, we conclude that egg overactivation triggers robust and expedited cell death by a calcium-dependent non-apoptotic mechanism.

## 2. Materials and Methods

### 2.1. Reagents

Water-soluble progesterone (PG), anesthetic MS-222 and ATP Bioluminescence Assay Kit CLS II were purchased from Sigma (St. Louis, MO, USA). hCG was from Teikoku Zoki (Tokyo, Japan) and collagenase (280 U/mg) was obtained from Wako (Osaka, Japan). The Hydrogen peroxide colorimetric/fluorometric assay kit was from BioVision (Milpitas, CA, USA). Fluorogenic caspase-3 substrate IV was purchased from Calbiochem (La Jolla, CA, USA). Polyclonal anti-cyclin B2 antibody was ordered from Santa Cruz (Santa Cruz Biotechnology, Dallas, TX, USA), biotinylated anti-rabbit IgG was from Vector Laboratories (Burlingame, CA, USA). The Streptavidin Biotin Complex Peroxidase Kit, protein assay CBB and hydrogen peroxide were from Nacalai Tesque (Kyoto, Japan). MitoTracker Deep Red FM was from ThermoFisher (Waltham, MA, USA). Luciferase control RNA and luciferase assay system were from Promega (Madison, WI, USA). Other chemicals were obtained from Wako and Nacalai Tesque. Slide glasses and cover slips for microscopy were purchased from Matsunami Glass (Osaka, Japan).

### 2.2. Animals and Cells

Adult wild-type female frogs *Xenopus laevis* were purchased from Shimizu (Kyoto, Japan) and maintained in dechlorinated water at the ambient temperature of 21–23 °C. The experiments with the animals were conducted according to the Kyoto Sangyo University Animal Experimentation Regulations under the permission N 2018–20. The experiments with oocytes and eggs were carried out at the ambient temperature of 21–23 °C. To isolate oocytes, the frogs were anesthetized in 2 mg/mL solution of MS-222, then the ovaries were surgically removed and placed into OR-2 solution containing 82.5 mM NaCl, 2.5 mM KCl, 1 mM CaCl_2_, 1 mM MgCl_2_, 1 mM Na_2_HPO_4_, 5 mM HEPES, pH 7.6. The ovaries were manually dissected into clumps of 50–100 oocytes and extensively washed with OR-2 solution. Oocytes were treated with 5 mg/mL collagenase in OR-2 at 21 °C for 3 h by shaking at 60 rpm, extensively washed in OR-2 solution and left for stabilization over 4 h. Undamaged defolliculated oocytes of stage VI, ranged in size from 1.2 to 1.3 mm, were manually selected and used in experiments. In vitro oocyte maturation was induced by addition of 5 mM PG and monitored by the appearance of a white spot on the animal hemisphere of oocytes. To obtain crude cytosolic fractions, eggs were homogenized by pipetting in tenfold volume of cold OR-2 buffer containing protease inhibitors APMSF and leupeptin and then centrifuged at 10,000 rpm, 4 °C, for 10 min. Supernatant fractions were collected and stored on ice until following biochemical analysis.

### 2.3. Microscopic Observations

Observation and imaging of *Xenopus* eggs were carried out using SZX16 stereo zoom microscope (Olympus, Tokyo, Japan) equipped with high-frame digital microscope CCD camera DP73, CCD interface U-TV0.5XC-3, wide-angle objective SDF PLAPO 1xPF. The CellSens Standard software (Olympus, Tokyo, Japan) was used for image acquisition. Acquired images were further processed with the ImageJ software of the National Institute of Health [29] freely available at https://imagej.nih.gov/ij/.

### 2.4. Treatment of Eggs with Hydrogen Peroxide and Calcium Chelators

Hydrogen peroxide was added at a final concentration of 10 mM to the oocytes matured in vitro for 10–12 h in the presence of progesterone. The cells were washed with OR-2 buffer before peroxide administration to remove the hormone. The precise concentration of hydrogen peroxide was determined by titration using the hydrogen peroxide colorimetric/fluorometric assay kit from BioVision, according to the manufacturer’s manual. To prepare 50 mM stock solutions of BAPTA (tetrasodium salt) and BAPTA-AM, the drugs were dissolved in water and DMSO, respectively. The chelators were added to eggs at a final concentration of 100 μM 30 min before hydrogen peroxide administration. At the dilution used, DMSO had no effect on egg viability. CaCl_2_ was excluded from the egg incubation media OR-2 in the experiments with calcium chelators.

### 2.5. Microinjections

Quantitative injections of luciferase mRNA into eggs and oocytes were made under microscopic observation with a pulse-directed injector system (Drummond, Nanoject). About 50 ng of the nucleic acid were injected in the oocyte cytoplasm. Control and microinjected eggs and oocytes were maintained in OR-2 media at 20 °C during the expression time of one hour. The individual gamete cells were analyzed immediately or frozen in liquid nitrogen and stored at −80 °C until luciferase detection.

### 2.6. Detection of Luciferase

The OR-2 incubation buffer was completely removed from the samples of individual oocytes and eggs, and a tenfold excess of the luciferase assay reagent from the luciferase assay system (Promega) was immediately added. The cells were disrupted by intense pipetting. Samples were clarified by pulse centrifugation, and their luminescence was measured for 10 s at room temperature, using a GeneLight GL-220 compact luminometer (Microtec, Funabashi, Japan).

### 2.7. Measurements of Intracellular ATP

To measure intracellular ATP contents, the ATP Bioluminescence Assay Kit CLS II was used according to manufacturer’s manual. Egg crude cytosolic fractions were obtained as described in Section 2.2. One-μL fraction aliquots were taken into 100-μL bioluminescence assays. Intensity of luminescence was quantified using the GeneLight GL-220 luminometer within one minute after initiation of luciferase reaction by sample addition.

### 2.8. Immunoblotting

To monitor cyclin B2 contents, crude cytosolic fractions of oocytes and eggs were heated at 95 °C for 5 min in the presence of SDS-sample buffer (62.5 mM Tris-HCl, pH 6.8, 2% SDS, 10% sucrose, 0.01% BPB, 100 mM DTT). Protein samples were separated by SDS PAGE using 10% polyacrylamide gels and transferred to PVDF membranes using a semidry blotting device from BioRad (Hercules, CA, USA). Membranes were blocked with T–TBS buffer (20 mM Tris–HCl, pH 7.5, 150 mM NaCl, 0.05% Tween 20) containing 3 mg/mL bovine serum albumin and incubated at room temperature for 2 h with a 200-fold diluted anti-cyclin B2 antibody. After washing with T-TBS buffer, the membranes were treated with the 1000-fold diluted biotinylated anti-rabbit IgG, then with the peroxidase-conjugated streptavidin, according to the manufacturer**’**s manual for the Streptavidin Biotin Complex Peroxidase Kit. The immune complexes were detected by color development catalyzed by peroxidase in the presence of hydrogen peroxide and diaminobenzidine tetrahydrochloride.

### 2.9. Other Methods

Protein content in egg cytosolic fractions was determined with the CBB protein assay. Sample absorbance was measured using a NanoDrop 1000 Spectrophotometer (Thermo Fisher Scientific, Waltham, MA, USA). Bovine serum albumin was utilized as a calibration standard. Caspase activity assay was performed as described previously [14]. Mitochondrial staining with the red fluorescent dye MitoTracker Deep Red FM were carried out as described previously [28]. Quantified data in figures are presented as means ± standard deviation (SD) values of four to six measurements taken in single-batch experiments. The experiments were repeated with the separate batches of eggs obtained from at least three different animals. From 50 to 100 eggs were observed in the experiments that concerned counting overactivated egg phenotype.

## 3. Results

### 3.1. Oxidative Stress-Induced Overactivation of Frog Eggs Involves Calcium-Dependent Mechanisms

**Figure 1:**
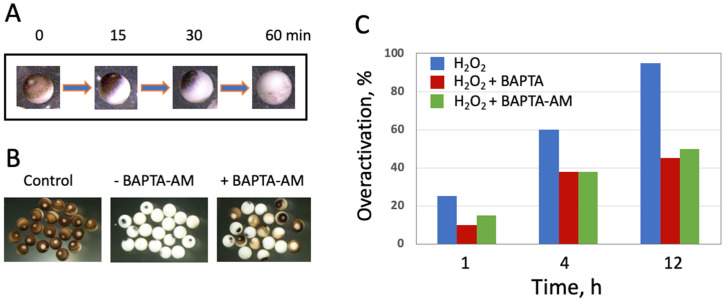
Involvement of calcium in the oxidative stress-induced overactivation of frog eggs. The progression of morphological changes in the overactivated eggs is presented in panel A. At time “0”, a population of *Xenopus* eggs was treated with 10 mM hydrogen peroxide, and a responsive (i.e., overactivated) egg was monitored over one hour following drug administration (panel (**A**)). Phenotypes of overactivated eggs treated with hydrogen peroxide for 12 h in the presence or absence of the selective calcium chelator BAPTA-AM (100 μM) are shown in panel (**B**). The rates of overactivation in the presence or absence of BAPTA or BAPTA-AM at different times are shown in panel (**C**). The experiment was repeated with three separate batches of eggs obtained from different animals and the result of a single-batch experiment is presented.

Oxidative-stress induced overactivation of *Xenopus* eggs leads to irreversible cortical contraction and complete egg whitening within 1 h (Figure 1A), in accordance with a previous report [28]. It is well established that multiple intracellular events, including cortical contraction, are triggered by an elevation of intracellular calcium in fertilized or parthenogenetically activated frog eggs. Moreover, the calcium signal represents an early indispensable event of fertilization-induced egg activation observed in all species studied [18]. Therefore, the involvement of intracellular calcium in egg overactivation was investigated. For this purpose, the selective calcium chelators BAPTA and BAPTA-AM were employed. The cell-permeable chelator BAPTA-AM greatly suppressed oxidative stress-induced cortical contraction at all observation times (1, 4, and 12 h), however the suppression was not complete. At all times, it affected only about 50% of eggs in the analyzed egg populations (Figure 1B,C). Unexpectedly, the cell-impermeable calcium chelator BAPTA exerted the same inhibitory effect on the oxidative stress-induced egg overactivation (Figure 1C), and the combined administration of both chelators did not increase the inhibition (data not shown), suggesting the same mechanism of action for the two drugs. In more detail, the similar response of peroxide-treated eggs to BAPTA and BAPTA-AM is debated in the “Discussion” section. In sum, our results indicate that intracellular calcium is involved in the oxidative-stress induced overactivation of *Xenopus* eggs. The data also disclose the existence of additional calcium-independent mechanisms of overactivation.

### 3.2. Degradation of Cyclin B Proceeds in the Absence of Caspase Activation in the Overactivated Eggs

**Figure 2:**
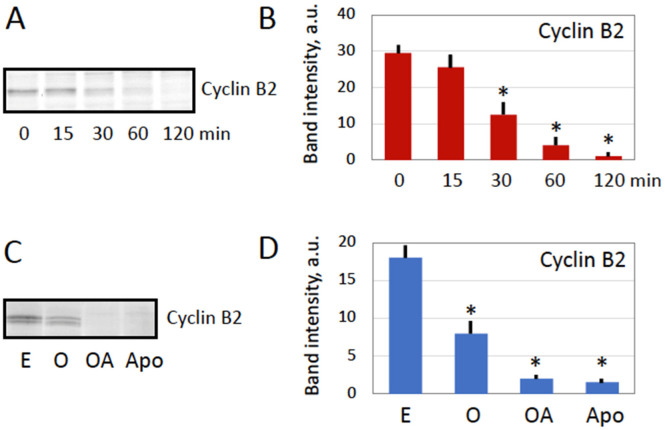
Cyclin B degradation in overactivated frog eggs. Cyclin B contents in the overactivated *Xenopus* eggs incubated with hydrogen peroxide for different times is presented in panels (**A**,**B**). Cyclin B contents in metaphase II-arrested (E), overactivated (OA), apoptotic (Apo) eggs and oocytes (O) are presented in panels (**C**,**D**). Panels (**B**,**D**) show quantification of the blots displayed in panels (**A**,**C**). In panels (**C**,**D**), overactivated eggs were collected after 1 h peroxide treatment, and apoptotic eggs were analyzed within 24*–*30 h of ovulation. The experiment was repeated with three separate batches of eggs obtained from different animals and the result of a single-batch experiment is shown. Bars in panels (**B**,**D**) represent SD values of the mean obtained in four measurements of the same experiment. Asterisks in the panels indicate statistical difference (*p* < 0.05) from untreated control eggs (0 min in panel (**B**), and E in panel (**C**)).

**Figure 3:**
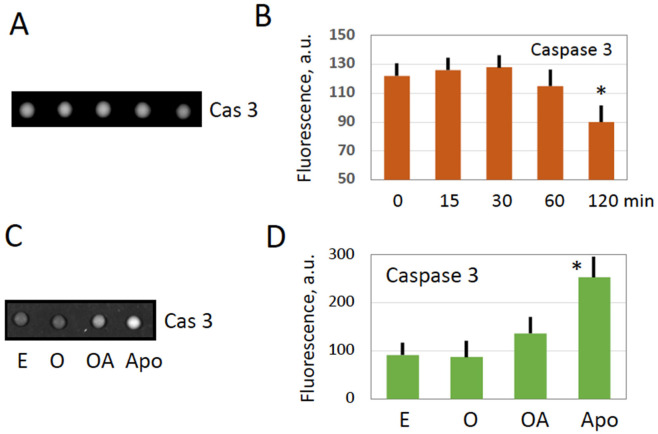
Caspase activity in overactivated frog eggs. Caspase 3/7 activity in the overactivated *Xenopus* eggs incubated with hydrogen peroxide for different times is presented in panels (**A**,**B**). Caspase activity in metaphase II-arrested (E), overactivated (OA), apoptotic (Apo) eggs, and oocytes (O) is presented in panels (**C**,**D**). Spot assays of caspase activity are shown in panels (**A**,**C**) and their quantification is presented in panels (**B**,**D**). In panels (**C**,**D**), overactivated eggs were collected after 1 h peroxide treatment, whereas apoptotic eggs were analyzed within 24–30 h of ovulation. The experiment was repeated with three separate batches of eggs obtained from different animals and the result of a single-batch experiment is shown. Bars in panels (**B**,**D**) represent SD values of the mean obtained in four measurements of the same experiment. Asterisks in the panels indicate statistical difference (*p* < 0.05) from untreated control eggs (E).

One of the hallmark biochemical events initiated by egg fertilization or parthenogenetic activation is calcium signal-mediated degradation of the M phase cyclin, cyclin B2, and the exit from meiotic metaphase II arrest. Considering that intracellular calcium is involved in oxidative stress-induced egg overactivation (Figure 1), it could be expected that cyclin degradation might occur in the overactivated eggs too. Indeed, it was found that cyclin B2 is rapidly degraded in the hydrogen peroxide-treated eggs, and the protein almost completely vanishes from the eggs within 60 min of the treatment (Figure 2A,B). Likewise, cyclin B was found to be completely degraded in the apoptotic frog eggs, and the reduced amount of the M phase cyclin was present in the immature prophase-arrested oocytes (Figure 2C,D). It has been reported previously that caspase-mediated apoptosis unfolds in unfertilized frog eggs following cyclin degradation and meiotic exit [14,15]. Activation of the main executive caspase, caspase 3, in apoptotic frog eggs was also confirmed in the present study (Figure 3C,D). However, in contrast to apoptotic eggs, no statistically significant elevation of caspase activity was observed in peroxide-treated overactivated eggs during one hour (Figure 3). Later on, caspase activity declined significantly in the overactivated eggs (Figure 3A,B) due to, presumably, all-out protein degradation that advances in these eggs after one-hour treatment [28]. Our findings highlight similarities and differences between overactivation-initiated cell death and apoptosis, as further discussed in the “Discussion” section.

### 3.3. Mitochondrial Function Is Suppressed in Overactivated Eggs

**Figure 4:**
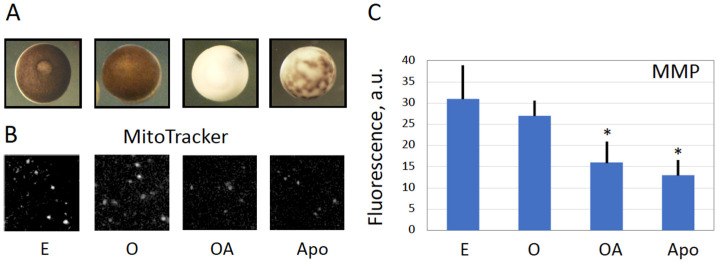
Decrease of the mitochondrial membrane potential in overactivated and apoptotic eggs. Morphological types of metaphase II-arrested (E), overactivated (OA), apoptotic (Apo) eggs and oocytes (O) are presented in panel (**A**). Corresponding fluorescent images of intracellular compartments, stained with the MitoTracker, are shown in panel (**B**). Panel (**C**) shows quantification of the data presented in panel (**B**). Fluorescent signals from more than ten individual mitochondria were evaluated in each cell type. Asterisks in panel (**C**) denote statistical difference (*p* < 0.05) from untreated control eggs (E).

Oxidative stress is known to induce mitochondrial stress that affects integrity and functional activity of mitochondria. Monitoring the mitochondrial membrane potential (MMP) is a common method to evaluate the functional state of mitochondria. In the present study, the mitochondrial potential-dependent red fluorescent dye MitoTracker Deep Red FM was employed to estimate the effect of egg overactivation on the functional activity of *Xenopus* egg mitochondria. Mitochondria from peroxide-treated eggs were compared to those from oocytes and untreated eggs. As a reference for mitochondrial disfunction, mitochondria from apoptotic eggs were analyzed too. The four different types of the cells can be readily distinguished by their characteristic morphology (Figure 4A). It was found that the fluorescent signal was significantly decreased in the overactivated peroxide-treated and apoptotic eggs, as compared to oocytes and untreated eggs (Figure 4B,C). This finding suggests that oxidative stress-induced overactivation of *Xenopus* eggs diminishes MMP and damages mitochondrial function. Notably, although the similar decrease in MMP was observed both in apoptotic and overactivated eggs, this phenomenon progressed much faster in overactivated eggs. It manifested just within one hour of triggering overactivation, however, it could be detected in apoptotic eggs only in about 24*–*30 h after ovulation [14], (Figure 4B,C).

### 3.4. Depletion of ATP and Termination of Protein Synthesis in Overactivated Eggs

**Figure 5:**
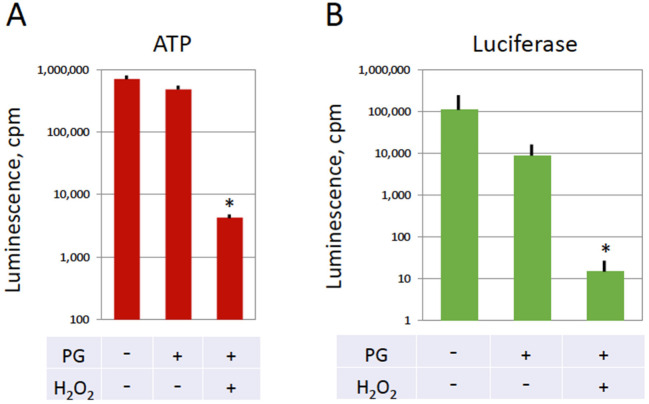
ATP content and protein synthesis in the frog eggs overactivated by oxidative stress. *Xenopus* oocytes were matured in vitro in the presence of progesterone and incubated in the presence or absence of hydrogen peroxide. Panel (**A**) shows the content of intracellular ATP in the overactivated eggs treated with 10 mM hydrogen peroxide for one hour and peroxide-untreated frog oocytes (PG−) and eggs (PG+), as evaluated by a chemiluminescent assay (see Section 2, “Materials and Methods” for details). Panel (**B**) presents the intensity of a luminescent signal generated by the luciferase protein synthesized in the oocytes and eggs after injection of luciferase mRNA. The luciferase reporter mRNA was microinjected into the cells immediately before hydrogen peroxide administration. Four to six oocytes or eggs were examined under each condition of treatment. Asterisks denote statistical difference (*p* < 0.05) from untreated control oocytes (PG−, H_2_O_2_−).

We have demonstrated previously that strong oxidative stress affects dramatically energy homeostasis in *Xenopus* eggs; nearly complete depletion of intracellular ATP was observed in the peroxide-treated eggs within 30 min of overactivation [28]. This finding was confirmed in the present study. A decrease in the intracellular ATP content of about two orders of magnitude was registered in the overactivated eggs after 1 h of hydrogen peroxide treatment (Figure 5A). It was also reported earlier that the content of soluble cytoplasmic protein gradually declines in the overactivated *Xenopus* eggs [28]. Notably, protein synthesis is one of the most energy consuming intracellular processes that requires both ATP and GTP for its execution. Therefore, the effect of overactivation on egg capacity to synthesize proteins was investigated. For this purpose, luciferase reporter mRNA was microinjected into the eggs followed immediately by hydrogen peroxide administration. Remarkably, synthesis of the luciferase protein was found to be effectively blocked in the peroxide-treated overactivated eggs; only a background level of the luciferase-generated luminescent signal could be observed in these eggs (Figure 5B). The detected level was about three to four orders of magnitude lower than that observed in control peroxide-untreated oocytes and eggs. The results obtained agree well with the rapid drop in intracellular ATP registered in the overactivated eggs, as presented in Figure 5A.

### 3.5. Overactivated Eggs Loose Plasma Membrane Integrity

**Figure 6:**
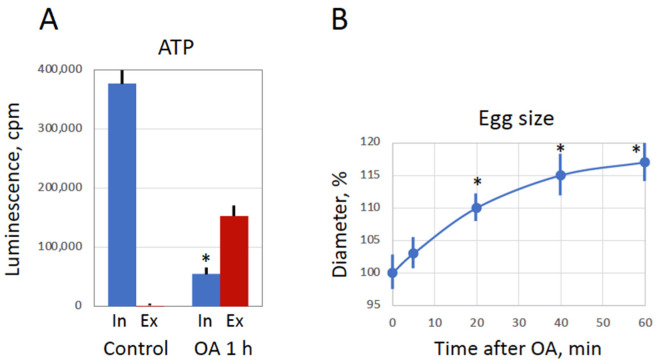
Breach of plasma membrane integrity in overactivated eggs. In vitro matured *Xenopus* eggs were treated with 10 mM hydrogen peroxide for one hour. Evaluations of intracellular (In) and extracellular (Ex) ATP in the cases of untreated (Control) and treated (OA 1 h) eggs are presented in panel (**A**). The progressive increase in the diameter of overactivated eggs is disclosed in panel (**B**). Panel (**A**) presents data of four to six ATP measurements in the same batch of eggs. More than ten eggs were analyzed in panel B at each time point. Asterisks denote statistical difference (*p* < 0.05) from untreated control eggs.

The extremely rapid and robust decline in intracellular ATP observed in overactivated eggs (Figure 5A) raised the question about possible mechanisms of the ATP depletion. It could be suggested that plasma membrane integrity was compromised in peroxide-treated overactivated eggs, thus allowing rapid ATP leakage from the cells. To examine this possibility, the contents of intracellular and extracellular, i.e., leaked, ATP were measured in the cases of untreated control eggs and overactivated eggs treated with hydrogen peroxide for one hour. It was found that significant amount of ATP leaked from the overactivated eggs during one hour of the treatment, while no or very little ATP was detected in the extracellular media of the control eggs incubated in the absence of peroxide during the same period (Figure 6A). As a result, overactivated eggs were almost completely depleted of intracellular ATP by the end of incubation. These data suggested that ATP depletion in overactivated eggs is associated with the loss of plasma membrane integrity. Accordingly, a significant increase in the diameter of overactivated eggs was also observed (Figure 6B), signifying the loss of cellular osmotic homeostasis regulated by membrane permeability Altogether, these results indicate that plasma membrane integrity is compromised in the overactivated eggs.

## 4. Discussion

**Figure 7:**
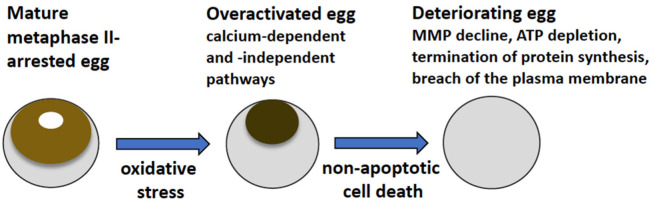
Oxidative stress-induced overactivation and deterioration of frog eggs. Strong oxidative stress triggers overactivation and cell death of mature metaphase II-arrested eggs of *Xenopus laevis* by a calcium-dependent non-apoptotic mechanism (see details in text).

The main findings of the present study are summarized in Figure 7. They reveal that strong oxidative stress initiates expedited death of mature, meiotically arrested frog eggs by a calcium-dependent non-apoptotic mechanism. These findings confirm our previous assumption that peroxide-overactivated *Xenopus* eggs degrade by a distinct non-apoptotic process [28]. Indeed, peroxide hydrogen-induced degradation of the overactivated eggs is extremely fast and robust. Its morphological features, such as irreversible cortical contraction, complete egg whitening and increase in egg diameter (Figure 1 and Figure 6), manifest clearly in the eggs within just one hour of triggering overactivation. In addition, multiple biochemical changes, such as cyclin degradation, decrease in MMP, depletion of intracellular ATP, termination of protein synthesis and breach of plasma membrane integrity (Figure 2, Figure 3, Figure 4, Figure 5 and Figure 6), occur in the eggs within one hour of peroxide treatment. In comparison, morphological features of apoptosis in aging unfertilized *Xenopus* eggs only become evident in about 18–24 h after ovulation [14].

Our data demonstrate clearly that peroxide-induced overactivation of frog eggs, resulting in their expedited death, is a calcium-dependent process. This conclusion is supported by several pieces of evidence. First, egg overactivation triggers cortical contraction (Figure 1A), which was shown to be calcium- and protein kinase C-dependent in fertilized or parthenogenetically activated *Xenopus* eggs [30,31,32]. Second, it was demonstrated previously that hydrogen peroxide elevates intracellular concentration of calcium and induces activation of *Xenopus* eggs via a Src kinase-dependent mechanism [27]. Third, egg overactivation can be potently inhibited by selective calcium chelators, as demonstrated in the present study (Figure 1). Finally, a downstream calcium-mediated event, such as degradation of the meiotic cyclin B2, was observed in peroxide-treated overactivated eggs (Figure 2). Although it is tempting to interpret cyclin degradation as the meiotic exit in overactivated eggs, additional studies, such as investigations of CSF activity and phosphorylation state of meiotically phosphorylated proteins, are required to confirm this conclusion.

It should be noted that although the involvement of calcium in egg overactivation is quite evident, some other calcium-independent mechanisms also should also contribute to this process. Indeed, the inhibition of egg overactivation by both cell-permeable (BAPTA-AM) and impermeable (BAPTA) selective calcium chelators, as well as by their combination, is only partial (Figure 1 and data not shown). It can be suggested that certain calcium-independent events of overactivation can still take place in the frog eggs treated with hydrogen peroxide in the presence of calcium chelators, whereas calcium-dependent events, such as cortical contraction, are blocked in these eggs. Further studies are necessary to reveal biochemical changes in these cells. The fact that both cell-permeable and -impermeable chelators are equally efficient in preventing peroxide-induced overactivation of frog eggs (Figure 1) can be explained in the light of the finding that plasma membrane integrity is compromised in overactivated eggs (Figure 6). It allows the cell-impermeable analog, BAPTA, to cross the damaged plasma membrane and inhibit intracellular calcium-dependent events of egg overactivation similarly to its call-permeable analog BAPTA-AM. It can be suggested that a drastic membrane breach in the peroxide-treated eggs occurs before intracellular calcium concentration reaches a threshold level that is necessary to trigger major calcium-dependent mechanisms. This would allow BAPTA to fully imitate the effect of permeable chelator BAPTA-AM, as presented in Figure 1. It should be noted in this connection that calcium release in fertilized or parthenogenetically activated *Xenopus* eggs is quite slow, it takes several minutes to reach its maximum [27]. In addition, the molecule of BAPTA is smaller than the molecule of ATP, suggesting that transmembrane diffusion of BAPTA may start earlier than ATP leakage from overactivated eggs (Figure 6A). Further studies are necessary to clarify the dynamics of membrane permeability change in overactivated frog eggs.

Next, our study reveals that the eggs overactivated by strong oxidative stress deteriorate by a process that is different from the classical apoptotic process previously described in meiotically arrested unfertilized frog eggs [14,15,33]. This conclusion is supported by several findings. First, morphological appearance of overactivated eggs is quite different from that of apoptotic eggs (Figure 4A). Although both cell death scenarios are preceded by intracellular calcium signal, the following calcium-dependent cortical contraction is reversible in the case of triggering apoptosis and irreversible in the case of overactivation, thus resulting in quite different cellular phenotypes. Second, the dynamics of overactivation-induced cell death is much faster than that of apoptosis. The dramatic detrimental changes in overactivated eggs, such as decrease in MMP, depletion of intracellular ATP, termination of protein synthesis and breach of plasma membrane integrity, occur in overactivated eggs just within one hour of triggering overactivation (Figure 3, Figure 4, Figure 5 and Figure 6). However, the events of the classical apoptosis, such as cytochrome C release, caspase activation, DNA fragmentation, decrease in the intracellular ATP content, etc. can only be observed in apoptotic eggs within 18–36 h after ovulation [14]. Third, in contrast to apoptotic eggs, no statistically significant caspase activation was observed in peroxide-treated overactivated eggs (Figure 3).

Oxidative damage was shown to disrupt mitochondrial function by lowering MMP, inhibition of respiratory chain and ATP production, and release of mitochondrial proteins into the cytoplasm [34]. Although it could be suggested that derangement of the mitochondrial membrane, as revealed by the decrease in MMP (Figure 4), might result in the release of mitochondrial apoptotic factors, such as cytochrome C, in the egg cytoplasm, caspase activation was found to be blocked in overactivated eggs (Figure 3). The reason for this may be the fast depletion of ATP observed in overactivated eggs (Figure 5). It is established that the decrease of intracellular ATP occurs quite late in the classical apoptotic process because high levels of ATP are required to maintain this process [35]. Specifically, apoptosome assembly, which is responsible for caspase activation, involves Apaf-1 and requires cytochrome C and ATP/dATP binding. It can be suggested that early depletion of ATP in overactivated eggs may prevent caspase activation by blocking apoptosome assembly. This suggestion requires experimental confirmation.

In the present study, egg overactivation was induced by a high concentration of hydrogen peroxide. This treatment provided a convenient biochemically tractable model of strong oxidative stress. However, the concentration of peroxide used in the study greatly exceeded its physiological levels. It was reported that the average intracellular concentration of hydrogen peroxide is about 10 nM, and the blood plasma concentration is about 100–5000 times higher, reaching micromolar and sub-millimolar concentrations [36]. Thus, although it seems unlikely that hydrogen peroxide is the major factor that triggers egg overactivation in vivo, it still can contribute to this process and work cooperatively in concert with other inducers of overactivation. In any case, the involvement of oxidative stress in deterioration of oocyte and egg quality is well documented. Oxidative stress was demonstrated to impair calcium homoeostasis, cause a decline in levels of critical meiotic regulators, such as MPF, induce mitochondrial dysfunction, and damage various intracellular macromolecules, such as DNA, proteins and lipids [37]. Notably, the physiological inducers of egg overactivation are currently unknown and they require identification.

In the end, it is established that the quality of frog eggs varies greatly, depending on the health and environmental conditions of the adult females producing the eggs [38]. Spontaneous activation has been implicated as a major factor responsible for the loss of fertilization capacity of ovulated eggs in many species, such as starfish, sea urchin, fish, frogs, etc. [18,39,40,41]. Spontaneous overactivation was also observed in populations of naturally ovulated frog eggs [28]. It occurs with a low frequency and is viewed as a pathological and uncontrollable process that makes egg fertilization impossible. Overactivated eggs can easily be recognized by their distinctive phenotype in aging populations of frog eggs (Figure 1 and Figure 4). Although the low frequency and spontaneous character of overactivation make difficult investigations of this process in the populations of naturally ovulated eggs, these studies should be carried out in the future. Identification of the physiological inducers of egg overactivation will help to attenuate this process, improve quality of eggs, delay their aging and eventually increase fertilization success. Considering a high degree of functional and physiological similarities between frog and mammalian eggs, the findings of frog studies can possibly be extended to mammals with applications in assisted reproduction. In addition, studies of overactivated eggs can broaden our understanding of cell death by revealing unexplored physiological mechanisms.

## 5. Conclusions

In the present study, strong oxidative stress was employed to induce overactivation and cell death of mature metaphase II-arrested eggs of *Xenopus laevis.* Our study demonstrates that (i) oxidative stress-induced overactivation of the eggs involves calcium-dependent mechanisms, and calcium-independent mechanisms also contribute to egg overactivation; (ii) cyclin B2 degradation, drop in MMP, ATP depletion and termination of protein synthesis occur in the eggs within one hour of triggering overactivation; (iii) plasma membrane integrity is compromised in the overactivated eggs; (iv) overactivated eggs degrade by a process that is different from the classical apoptosis. Further studies are necessary to identify physiological inducers of egg overactivation and reveal factors that can attenuate this process and improve egg quality.

## Data Availability

Data is contained within the article.
